# Integrative Single-Cell RNA-Seq and ATAC-Seq Analysis of Peripheral Mononuclear Cells in Patients With Ankylosing Spondylitis

**DOI:** 10.3389/fimmu.2021.760381

**Published:** 2021-11-22

**Authors:** Huixuan Xu, Haiyan Yu, Lixiong Liu, Hongwei Wu, Cantong Zhang, Wanxia Cai, Xiaoping Hong, Dongzhou Liu, Donge Tang, Yong Dai

**Affiliations:** ^1^ Clinical Medical Research Center, Guangdong Provincial Engineering Research Center of Autoimmune Disease Precision Medicine, Shenzhen Engineering Research Center of Autoimmune Disease, The Second Clinical Medical College of Jinan University, Shenzhen People’s Hospital, Shenzhen, China; ^2^ Department of Nephrology, The First Affiliated Hospital of Jinan University, Guangzhou, China; ^3^ Guangxi Key Laboratory of Metabolic Diseases Research, Guilin Key Laboratory of Kidney, Diseases Research, 924st Hospital, Guilin, China

**Keywords:** ankylosing spondylitis, single-cell RNA sequencing, single-cell assaying transposase accessible chromatin sequencing, NFkB, TNF signaling pathway

## Abstract

**Objective:**

Genetic studies on ankylosing spondylitis (AS) have identified more than 100 pathogenic genes. Building a bridge between these genes and biologically targeted therapies is the current research hotspot.

**Methods:**

We integrated single-cell assaying transposase-accessible chromatin sequencing (scATAC-seq) and single-cell RNA sequencing (scRNA-seq) to explore the key genes and related mechanisms associated with AS pathogenesis.

**Results:**

We identified 18 cell types in peripheral mononuclear cells from patients with AS and normal controls and summarized the cell-type-specific abnormal genes by scRNA-seq. Interestingly, we found that the pathogenic gene *NFKB* involved in AS progression originated from CD8+ T cells. Moreover, we observed an abnormal tumor TNF pathway mediated by abnormal expression of *TNF*, *NFKB*, *FOS*, *JUN*, and *JUNB*, and scATAC-seq results confirmed the abnormal accessible binding sites of transcriptional factors *FOS*, *JUN*, and *JUNB*. The final magnetic bead sorting and quantitative real-time PCR(RT-qPCR) confirmed that *NFKB*, *FOS*, *JUN*, and *JUNB* in CD8+ T cells differed in the AS group.

**Conclusions:**

Our results revealed a possible mechanism by which *NFKB* abnormally regulates *FOS*, *JUN*, and *JUNB* and drives *AS* progression, providing a novel perspective from a single cell point of view in AS.

## Introduction

Ankylosing spondylitis (AS) is an immune-mediated spondyloarthropathy (1). AS is difficult to cure and requires lifelong treatment, and causes a decline in the quality of life (2, 3). AS genetics has made exciting progress by discovering more than 100 genetic variants that influence disease risk (4–6). However, these genetic research results are still difficult to apply to the targeted therapy of AS (7). The problem of how to translate genetics into new biology and drug targets remains to be resolved. The first step in solving this problem is to determine the relevant cell type (s) in which causal genes exhibit their function(s). Next, we need to determine how causal genes play a role in the cell environment.

Single-cell high-throughput technology allows scientific research to enter the single-cell era at the population level (8). Single-cell RNA sequencing (scRNA-seq) allows researchers to capture the transcription status of each cell (9), while assaying transposase-accessible chromatin in single-cell sequencing (scATAC-seq) can reveal biological processes through the degree of chromatin openness (10). Combining scRNA-seq with scATAC-seq allows researchers to identity pathogenic genes and their associated cell types (8, 11). More importantly, single-cell technology will allow us to study the cellular background of the pathogenesis of disease more rigorously and validate therapeutic targets functionally (12).

The human blood immune system plays a vital role in the progression of autoimmune diseases. Peripheral blood mononuclear cells (PBMCs) are composed of various immune cells (13) that participate in the progression of various immune activities and the occurrence of inflammatory reactions. AS is a polygenic genetic disease caused by genetic and environmental factors (14). Immune cells play an crucial role in pathogenesis (15). Our study provides a high-resolution transcriptional and chromatin accessibility map of AS causal genes by combining scATAC-seq and scRNA-seq analysis, which vividly shows the process of causal genes participating in AS and the detailed functional interactions between immune cells. A study on the scRNA-seq of AS’s PBMCs has been reported previously (16), and previous research has focused on monocytes and natural killer (NK) cells. Our study focused on how T cells participate in the pathogenesis of AS. This will enrich the data of AS single-cell research and provide another perspective for understanding the pathogenesis of AS. Moreover, it will provide a more intuitive and novel perspective for transforming AS genetic results into drug-targeted therapy.

## Results

### Cell−Type−Specific Clustering by scRNA-Seq

**Figure 1:**
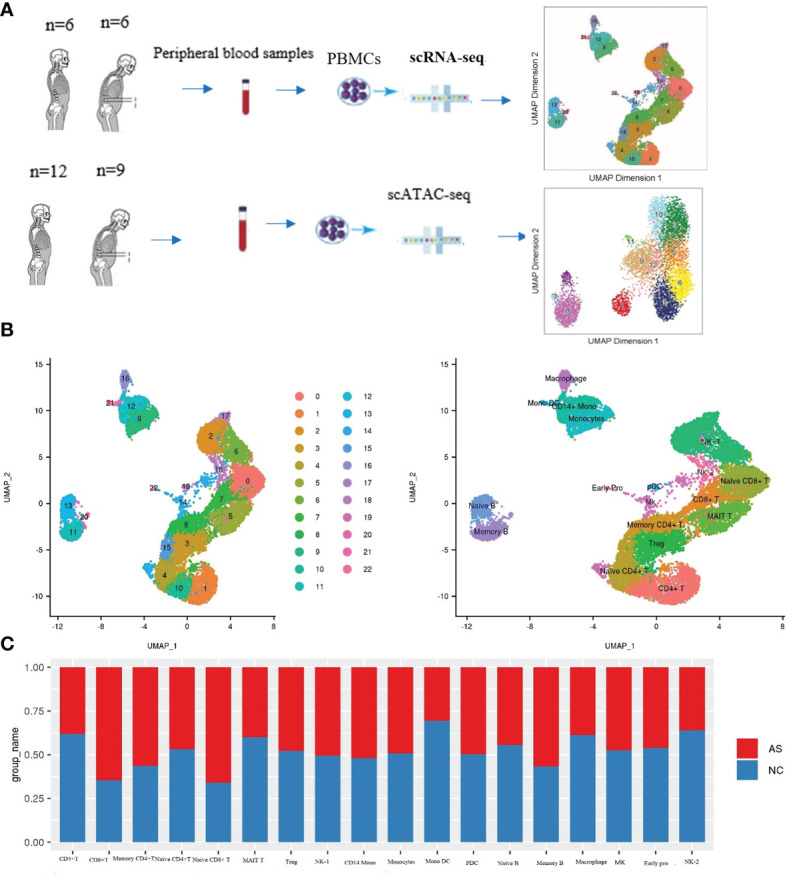
Cell-type-specific clustering and analysis of human PBMCs according to ScRNA-seq. **(A)** Workflow of single-cell RNA-seq and single-cell ATAC-seq of the PBMC of the patients with AS and normal controls (NC); **(B)** UMAP plots showing 23 leukocytic clusters corresponding to 18 cell types according to known cell marker genes through SCRNA-seq; **(C)** Cell abundance in each cell type across the AS-PBMC and NC-PBMC libraries.

**Figure 2:**
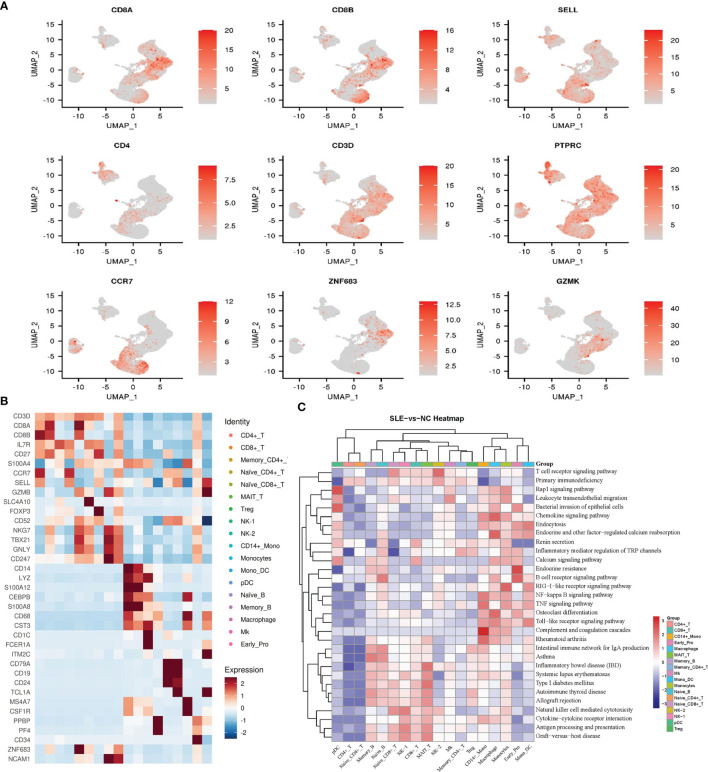
Cell type identification and functional analysis of differential genes between the AS-PBMC and NC-PBMC. **(A)** UMAP plots of canonical cell markers used to identify clusters, color-coded for expression levels. **(B)** Heatmap to show the expression levels of the selected gene markers of each cell type. **(C)** Pathway enrichment analysis (KEGG) of significantly differential gens between the AS-PBMC and NC-PBMC libraries (p < 0.05). Heatmap expression is the result of Z-score column-wise normalization. The P-value is represented by the color. All P values are less than 0.05. The redder the color, the smaller the P value, indicating that the enrichment was more notable. The white color represents that DEGs of this cell type is not enriched in this signaling pathway.

To obtain single-cell transcriptional profiles of AS, we isolated and re-clustered the mononuclear cells from the peripheral blood of six AS patients with different disease courses and disease severity. Single-cell transcriptional profiles of the NC group paired with AS were also obtained (**Figure 1A**). For the raw sequence data, we used Cell Ranger software to obtain the gene expression matrix and used Seurat software (11) for further analysis. After quality filtering, 16,618 cells were considered to be high-quality cells. Of these, 7,665 cells (46.12%) from the AS-PBMC and 8,953 cells (53.88%) (**Supplementary Table 1**). Unsupervised clustering analysis using the Seurat software identified 18 distinct cell types from PBMCs in AS-PBMC and NC-PBMC libraries after scRNA-seq analysis. These identified cell clusters based on marker genes could be readily assigned to known cell lineages. In detail, there were naïve CD8+T cells (Cluster 0), NK-1 cells (Cluster 2, 6 and 17), Treg cells (regulatory cells) (Cluster 3), naïve CD4+T cells (Cluster 4 and 15), MAIT T cells (Cluster 5), CD8+T cells (Cluster 7), memory CD4+T cells (Cluster 8), monocytes (Cluster 9), CD4+T cells (Cluster 1 and 10), memory B cells (Cluster 11 and 20), CD14+ monocytes (Cluster 12), naïve B cells (Cluster 13), megakaryocyte progenitor (Cluster 14), macrophages (Cluster 16), NK-2 (Cluster 18), PDC (plasmacytoid dendritic cells) (Cluster 19), monocyte-derived dendritic cells (Cluster 21), and early progenitor/endothelial cells (Cluster 22), as shown in **Figure 1B**. The mark used to identify the cell type is shown in the figure with a heatmap. Of the mature blood cell types, we identified CD8+T cells (expressing *CD8*+, *CD8A*+,and *CD 8B*+), naïve CD8+ T cells (expressing *CD8*+, *CD8A*+, *CD8B*+, *GZMK*+, and *TNFSF8*+), CD4+T cells (expressing *CD4*+), naïve CD4+T cells (expressing *CD4+* and *SELL+*), memory CD4+T cells (expressing *CD4*+, and *IL7R*+), etc. (**Figures 2A, B**, and **Supplementary Table 2**).

Interestingly, we found a difference in the cell type frequencies between AS and NC, so we performed a statistical analysis to compare cell frequencies. The percentage of different cell types in AS-PBMC and NC-PBMC is shown in **Figure 1C**. Cell clusters that were significantly over-represented in the AS-PBMCs included naïve CD8+T cells, CD8+T cells, memory CD4+T cells, and memory B cells. Among them, the difference in the proportion of the AS group in CD8+T cells and naive CD8+ T cells was particularly significant at 61% and 62%, respectively. Cell clusters significantly over-represented in the NC-PBMCs included MAIT T cells, CD4+T cells, mono DCs, naïve B cells, macrophages, and NK-2. There was no significant difference in the distribution of the remaining clusters between the two groups. Therefore, scRNA-seq analysis uncovered the heterogeneity of cell clusters in AS-PBMCs. In terms of cell ratio, the overexpression of CD8+ T cells and naive CD8+ T cells in AS may suggest that CD8+ T cells play an important role in the development of AS.

### Bioinformatics Analysis of AS-PBMC and NC-PBMC Libraries Reveal the Gene Regulatory Network by scRNA-Seq

To identify the key genes involved in the pathology of AS, we identified differentially expressed genes (DEGs) in AS compared with controls in each cell type. In our study, there were a total of 279 DEGs between different cell clusters in the AS and control groups. Further Gene Ontology (GO) and Kyoto Encyclopedia of Genes and Genomes (KEGG) analyses were performed for each cell type, and the functional enrichment results showed that most of these DEGs were involved in the pathophysiological processes related to immunity and calcium metabolism (**Figure 2C** and **Supplementary Figures 1**, **2**).

The GO analysis indicated that DEGs in CD8+ T cells were related to biological regulation, regulation of biological processes, and immune responses, and enriched 30 pathways in the KEGG pathway enrichment analysis. The enriched pathways include antigen processing and presentation, TNF signaling pathway, and so on. Previous studies have reported that these signaling pathways (17) are related to immune diseases and are directly related to AS.

**Figure 3:**
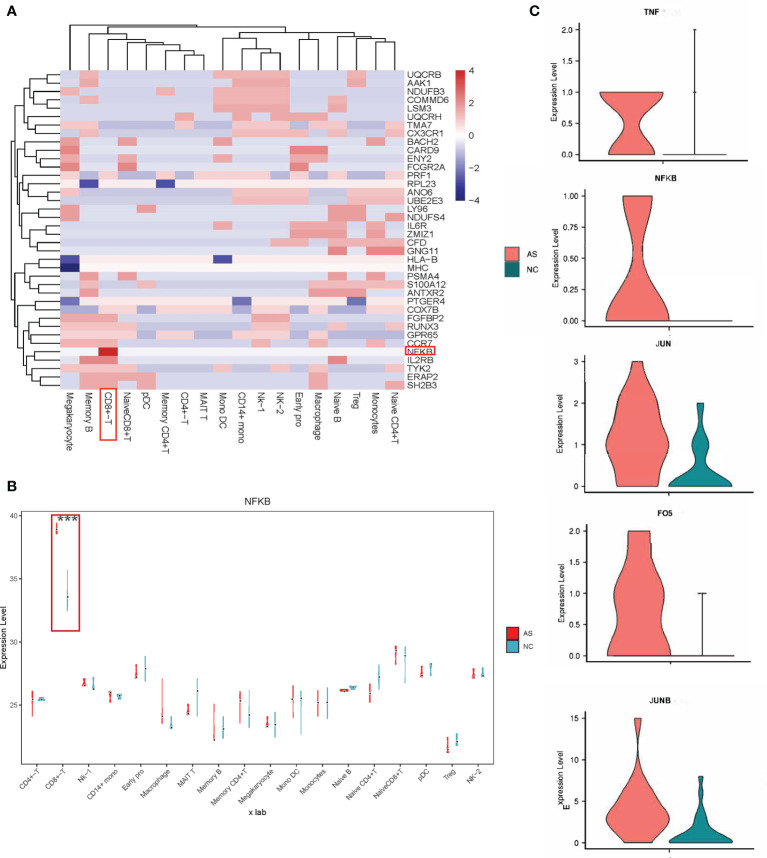
Relationship between pathogenic genes and specific cell types. **(A)** Heatmap representing the AS causal genes expression levels in the specific cell type. The scale bar displays fold change values; Fold change value “0” is given for genes which are not DE, statistical significance was taken at P values of less than 0.05 (P < 0.05). and a fold-change criterion (FC>1.2); **(B)** Violin-box plots for NFKB expression level by cell type, compared to respective controls. The results for AS are shown in red, and those for NC are in green; ***p < 0.001. **(C)** Violin plots comparing the expression of selected genes (*TNF, NFKB, JUN, JUNB, FOS*) in CD8+T cells across AS and NC. The results for AS are shown in red, and those for NC are shown in green.

To date, dozens of pathogenic genes associated with AS development have been identified. We used the meta-analysis method to identify pathogenic genes related to AS pathogenesis (4, 5, 18, 19). We overlapped these pathogenic genes with the DEGs identified in each cell type in our study. As a result, we determined the cell types related to these pathogenic genes based on our single-cell transcriptome data (**Figure 3A**). Associating disease-causing genes with specific cell types allows us to have a more three-dimensional and comprehensive understanding of how these genes participate in the occurrence and development of AS.

### Locate the Most Relevant Cell Type and Cell-Type-Specific Genes for AS by scRNA-Seq

As shown in **Figure 3B**, *NFKB* differentially expressed between AS and NC had the most significant statistical significance in CD8+ T cells. Many studies have shown that increased expression of *NFKB* is closely related to the pathogenesis of AS (20–22), but the detailed mechanism remains unclear. In this study, the proportion of CD8+ T cells in AS was significantly increased, consistent with previous studies showing that CD8+ T cells play a role in the progression of AS (23–28).

**Figure 4:**
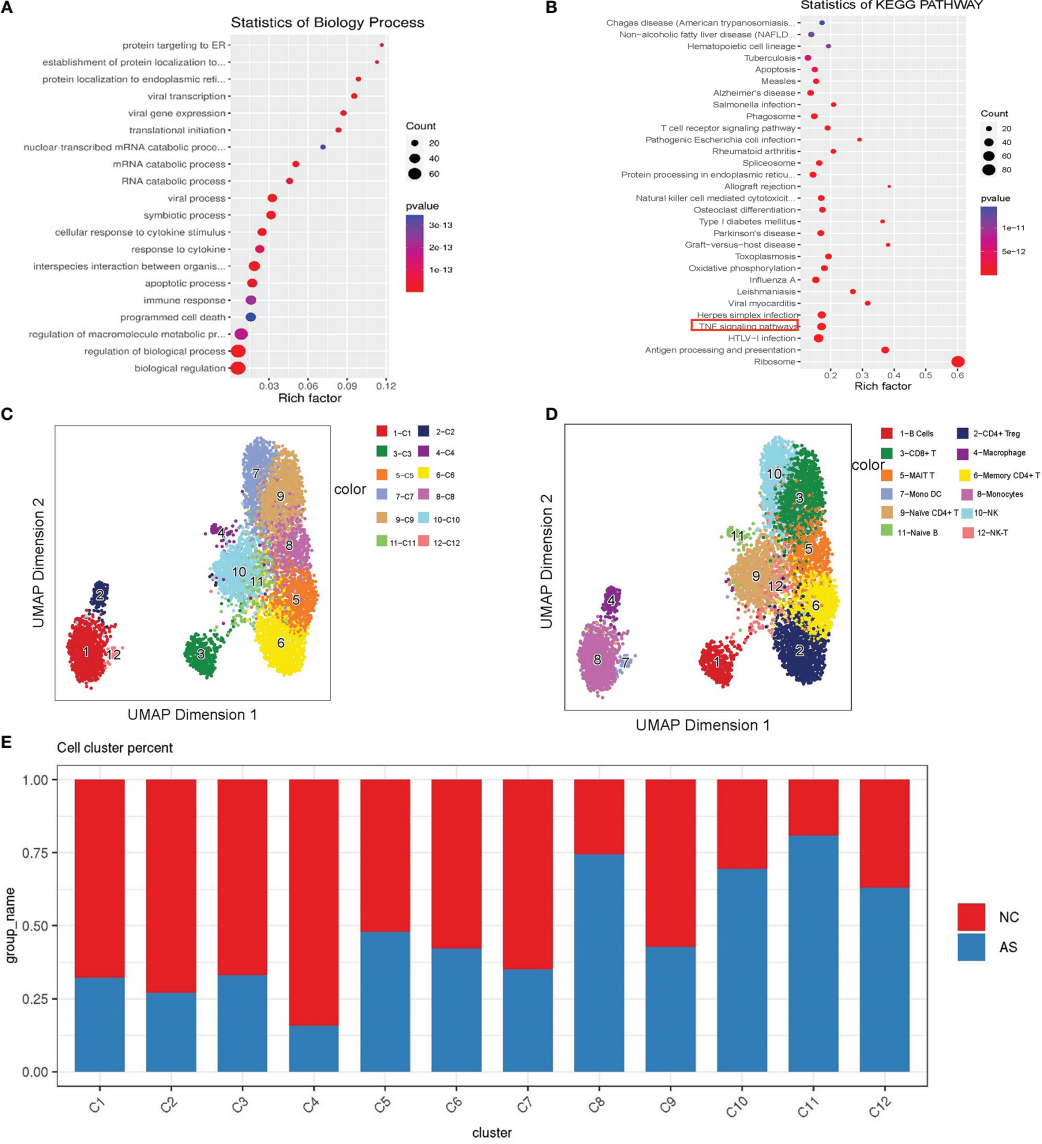
Single-cell analysis of CD8+T cell. **(A)** GO term analysis for biological processes of differentially differential genes between the AS-PBMC and NC-PBMC scRNA-seq libraries in CD8 +T cells (p < 0.05); **(B)** KEGG pathway analysis of differentially differential genes between the AS-PBMC and NC-PBMC scRNA-seq libraries in CD8 +T cells (p <0.05). The red border indicates the TNF signaling pathway; **(C)** A UMAP plot showing 12clusters through SCATAC-seq; **(D)** A UMAP plot showing 12 leukocytic clusters corresponding to 12 cell types according to known cell marker genes through SCATAC-seq; **(E)** Cell abundance in clusters across the AS-PBMC and NC-PBMC libraries.

Our results showed that *NFKB* in CD8+ T cells participates in 11 signal pathways (**Figures 4A, B**), including the TNF signaling pathway, apoptosis signaling pathway, and hepatitis B signaling pathway. Interestingly, abnormal TNF signaling in CD8+ T cells has been widely reported to play a vital role in AS pathogenesis (29–32). We found a series of differentially expressed genes in a mutually regulated signal chain in the TNF signal pathway (**Figure 3C** and **Supplementary Figure 3**). This group of genes is distributed in both the upstream and downstream of the signal pathway, regulating and influencing each other. This signal chain includes *TNF, NFKB, FOS, JUN*, and *JUNB*. Notably, *FOS, JUN, JUNB* are also transcription factors.

### Labeling scATAC-Seq Clusters With scRNA-Seq Information

For the analysis and verification of this regulatory network with genes and transcription factors, scATAC-seq analysis is a suitable method. Our group members have already recruited nine patients with AS and 12 healthy volunteers to perform scATAC-seq analysis in published articles (33). To integrate the data of scRNA-seq and scATAC-seq for correlation analysis and obtain further detailed information, we re-analyzed the original scATAC-seq data. After quality control, we identified the clusters and predicted cell types based on scRNA-seq. As shown in **Figure 4C**, we annotated 12 cell types according to the known cell marker genes. These included the following cell types: CD8+T cells, B cells, monocytes, CD4+Treg cells, memory CD4+T cells, macrophages, naive B cells, NK-T cells, MAIT T cells, naive CD4+ T cells, and NK cells (**Figure 4D**).

Similarly, we found that the proportions in each cluster are different. Statistical tests revealed significant differences among the cell types. AS-PBMCs account for a larger proportion of some cell types, including CD8+T cells, B cells, monocytes, memory CD4+T cells, macrophages, naïve B cells, and NK cells. In AS-PBMCs, CD8+T cells account for 78%. This is consistent with the scRNA-seq analysis results. This further confirmed the heterogeneity of CD8+T cells in the AS-PBMCs (**Figure 4E**).

### Single-Cell Chromatin Accessibility of CD8+ T Cell

**Figure 5:**
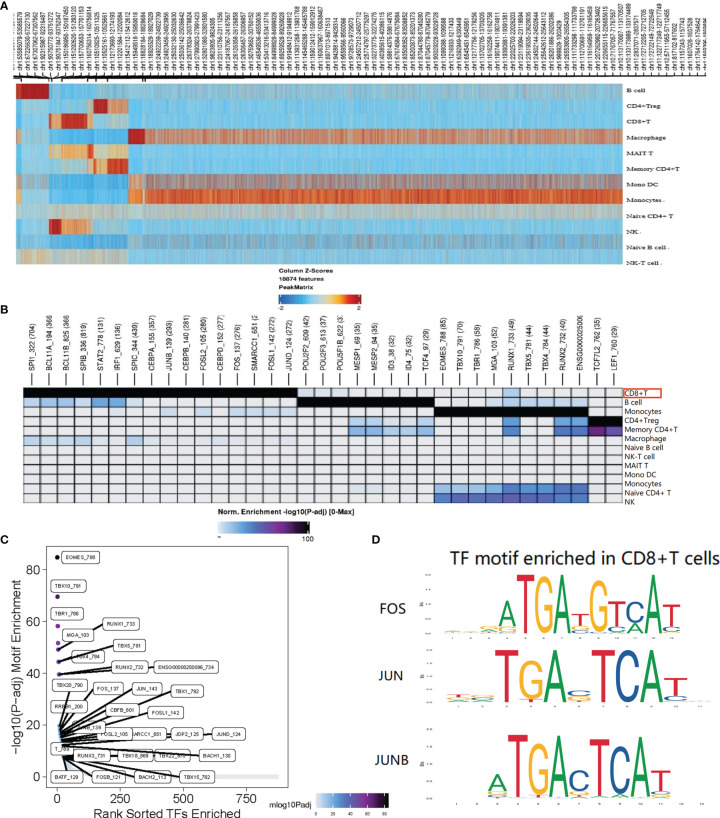
Single-cell ATAC analysis of CD8+T cell. **(A)** The heatmap shows the peaks that are specific for each cell type. **(B)** Different TF motifs in each cluster are specific for each cell type (p < 0.05); The red border indicates the motif associated with genes *FOS*, *JUN*, and *JUNB*; **(C)** CD8+T cell specific motif highly enriched in the peak; **(D)** *FOS*, *JUN*, and *JUNB* with differential accessibility between the AS-PBMC and NC-PBMC libraries across CD8+T cell. P values shown in this figure were calculated with ArchR through the difference analysis feature and adjusted using the Benjamini–Hochberg correction for multiple tests.

A total of 1152 cells were detected in the CD8+ T cell cluster, of which 904 cells were derived from AS-PBMCs, and the remaining 248 cells were derived from NC-PBMC. There were 843 differentially expressed peaks in the CD8+T cells (**Figure 5A**). With transcription factor motif analysis using ArchR, we found that *JUN*, *JUNB*, and *FOS* binding sites had differential accessibility in CD8+ T cells in the AS group. The differential accessibility of these TF binding sites was considered as crucial for the TNF signaling pathway. These *TNF-NFKB* signaling motifs were more enriched in CD8+T cells than in other cell types in the AS group (**Figures 5B**–**D**). This further illustrates that *TNF-NFKB-FOS*, *JUN*, and *JUNB* in CD8+ T cells may participate in AS through the TNF signaling pathway.

Yu (33) in our research team used a different clustering method (the method can be seen at: https://support.10xgenomics.com/single-cellatac/sofware/pipelines/latest/algorithms/overview) to analyze the scATAC sequencing library from 12 normal controls (NCs) and nine patients with AS. Among the seven different functional cell types, including NK cells, monocytes, memory CD4+ T cells, CD8+ T cells, B cells, and DCs, it was found that CD8+ T cells play an essential role in AS pathogenesis. In the data processing and analysis conducted by Dr. Yu, they found in the TF motif analysis that these motifs in CD8+ T cells of AS are more highly enriched than those in CD8+ T cells of NC. This again verifies the experimental results.

### Validation of Key DEGs by Immunomagnetic Bead Sorting and Quantitative Real-Time Polymerase Chain Reaction (RT-qPCR)

**Figure 6:**
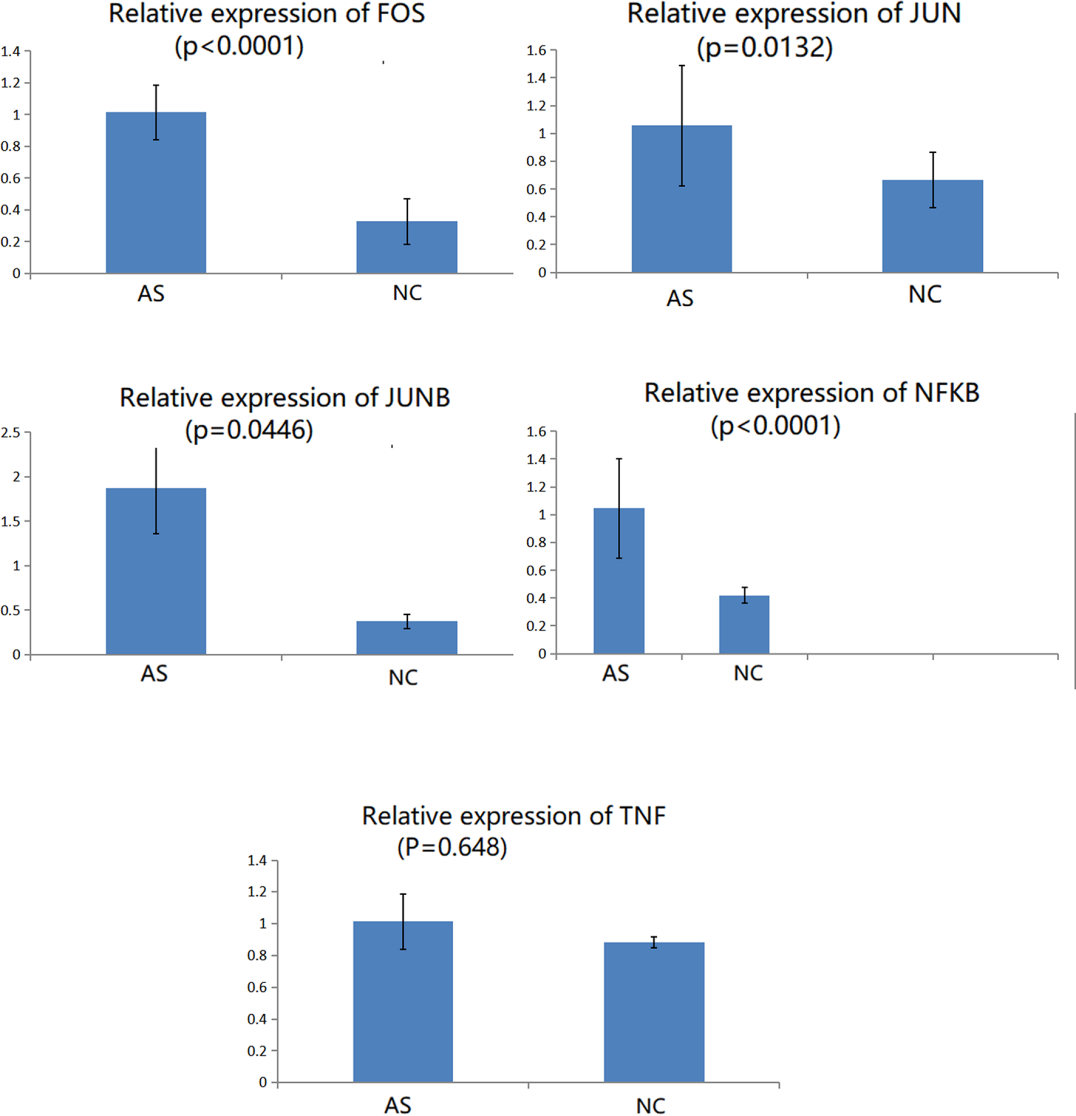
Relative expression of genes in CD8+ T cells involved in the TNF signaling pathway.

CD8+T cells were purified by magnetic bead cell sorting. We further checked the expression of five genes in CD8+T cells (T*NF, NFKB, FOS, JUN*, and *JUNB*). Four genes were confirmed to be upregulated in AS with statistically significant differences: *NFKB, FOS, JUN, and JUNB* (**Figure 6**). In the subsequent RT-qPCR analysis of *TNF*, there was no statistically significant difference between the CD8+ T cells of NC and AS (P=0.648).

## Discussion and Conclusions

AS is an inflammatory disease characterized by affecting the sacroiliac joints and axial skeleton (14). At present, the mining of AS pathogenic genes cannot be substituted into the cellular environment to understand the pathogenic process. This affects the development of targeted drugs. Traditional bulk RNA-seq detects the average expression of genes from multiple cells, which naturally masks the signal of cell heterogeneity, especially for some signals from rare cell subpopulations due to neutralization (34). Meanwhile, scRNA-seq can detect such differences and improve the resolution of gene expression. Thus, we used scRNA-seq coupled with meta-analysis to detect differential expression of specific cell subsets in PBMCs, and infer specific cell types corresponding to AS-causing genes by clustering expression profiles. However, there are two limitations of this approach that should be considered. First, some of the included genes have not passed the functional verification, and more functional experiments in the future are needed for verification. Second, although these genes are pathogenic genes of AS, they are not all directly involved in pathological processes in the PBMCs of patients with AS. Therefore, the included genes need a larger sample to vertify the universality and specificity.

AS is an autoimmune disease involving many types of immune cells (35). Research on CD8+T cells is believed to mediate their pathogenicity through several different mechanisms. Cytotoxic T lymphocytes induce direct lysis of target cells. In addition, CD8 effector T cells secrete inflammatory cytokines, including TNF-α, IFN-γ, and IL-17. Furthermore, CD8+T cells support a chronic immune response in patients with AS (36). We observed abnormal expression of *TNF*, *NFKB*, *FOS*, *JUN*, and *JUNB* in CD8+ T cells, and these genes were enriched in the TNF signaling pathway. The abnormal TNF signaling pathway in CD8+T cells plays a role in the pathogenesis of AS (37). The clinical application of anti-TNF therapy has changed the treatment of chronic inflammatory diseases, such as AS. However, not all patients are sensitive to TNF inhibitors (TNFi) (38). TNFi is recommended as the first biological treatment for AS and non-radiographic axial spondyloarthritis (2). The reasons for the failure of TNFi therapy need to be explained from a biological perspective.

Our scRNA-seq analysis and scATAC-seq analysis provide a rich data source for discovering *TNF, NFKB, FOS, JUN*, and *JUNB* genes that might play a role in CD8+ T cells in the TNF signaling pathway. The final magnetic bead sorting and RT-qPCR confirmed that *NFKB, FOS, JUN*, and *JUNB* in CD8+ T cells differed in the AS group. Since *TNF* acts as an external ligand in the TNF signaling pathway, we speculate that there may be increased secretion of *TNF* from other cells for binding to the TNFR receptor in CD8+ T cells, followed by the activation of the TNF signaling pathway. *NFKB*, an important factor for inducing the expression of many proteins, has been identified as a promoter and enhancer of many genes. These genes expresses cytokines, acute-phase response proteins, and cell adhesion molecules, which enhance the immune response (39). As the innate immune response center coordinator, *NFKB* is involved in the occurrence and progression of many autoimmune diseases. In this study, *NFKB* was specifically upregulated in the PBMCs of the AS group. The corresponding upstream genes, *TNF*, encoding cytokines and downstream transcription factors *FOS, JUN*, and *JUNB* were also upregulated. Notably, ‘prop. test’ other than ‘scProportion Test’was used for comparing cell type ratios. The method of scProportion Test just appeared since 2021 (https://github.com/rpolicastro/scProportionTest) (40, 41). This R library facilitates the analysis of the difference between the proportions of cells in clusters between two scRNA-seq samples. A permutation test is used to calculate the p-value for each cluster, and a confidence interval for the magnitude difference is returned *via* bootstrapping. There is also a function to generate a point range plot to display the results.Although prop. test focuses more on obtaining the confidence interval of the probability parameter, this method was the most suitable and most recognized method for discrete data of cell ratio comparison before 2021 (42–44).

Moreover, our scATAC-seq data confirmed the abnormal accessibility of *FOS*, *JUN*, and *JUNB*, which is consistent with a previous report (33). Interestingly, *TNF*, *NFKB*, *FOS*, *JUN*, and *JUNB* can construct a signaling pathway with upstream and downstream regulation. Notably, the FOS gene family encodes leucine zipper proteins, which can dimerize with the Jun family proteins to form a transcription factor complex AP-1 (45). AP-1 has been shown to be involved in inflammation and immune responses (46). AP-1 directly participates in the pathogenesis of AS downstream of the signaling pathway. Therefore, we can speculate that *NFKB* can trigger abnormal TNF signaling pathways by regulating *FOS*, *JUN*, and *JUNB*, thereby promoting the development of AS. Our results emphasize that *NFKB* acts as a regulator of the immune response process and participates in the pathogenesis of AS. Our findings provide new insights into the biology of the action of TNF blockers from an etiological point of view. They can further provide relevant signal transduction pathways for new biological therapies. Notably, AS mainly affects the axial joints of the human body. Our research on PBMCs in patients with AS may not display the exact pathophysiological changes in the axial joints. Since AS is an autoimmune disease, and the immune cells circulate in the body through blood, research on PBMCs contributes to the identification of markers for diagnosis and targeted therapy. For example, a recent study (47) on PBMC of AS patients has mentioned that granulocyte macrophage-colony stimulating factor (GM-CSF) could be detected in plasma from 14/46 (30%) AS patients compared to 3/18 (17%) healthy controls, and GM-CSF neutralization can be a potential novel therapeutic approach for the treatment of AS.

In conclusion, we associate AS pathogenic genes with specific cell types at single-cell high resolution. This provides a new perspective for subsequent research on AS pathogenesis. Furthermore, we suggest that *NFKB* regulating *FOS*, *JUN*, and *JUNB*, and causing an abnormal TNF signaling pathway, may be an important factor in the progression of AS. Therefore, the development of specific inhibitors of these mutually regulated genes and their protein products may lead to novel therapeutics and create a new basis for the biological treatment of AS.

## Materials and Methods

### Study Design

This study was approved by the Ethics Committee of Jinan University and was conducted according to the principles of the Declaration of Helsinki. All participants provided informed consent and signed a consent form. Blood samples were obtained from patients fulfilling the AS diagnosis according to the modified New York criteria (48). AS disease activity was assessed using the AS Disease Activity Score and C-reactive protein (ASDAS-CRP) (49). The exclusion criteria included AS along with other immune diseases, arthritis or systemic lupus erythematosus; AIDS or other immunodeficiencies; using high-dose (>1 mg/kg/day) glucocorticoids and other immunosuppressants; and pregnancy. The blood samples of scRNA-seq from AS subjects (n = 6, male/female = 6/0, mean age 27 ± 6 years) and healthy controls (n = 6, male/female = 6/0, mean age 24 ± 3 years) were recruited from outpatient clinics or medical staff at Shenzhen People’s Hospital in 2019 (Shenzhen, China). The AS and NC scATAC-seq samples were obtained from nine outpatients in the rheumatology clinic and 12 healthy age- and sex-matched volunteers in Shenzhen People’s Hospital in 2019. The characteristics of the enrolled patients with AS and NCs are shown in **Supplementary Table 3**.

### Library Construction of Single-Cell Libraries

After PBMC isolation, we used 10X Genomics (50) to establish single-cell sequencing libraries. After the library was established, a Bioanalyzer (Agilent) was used for quality control. The KAPA Library Quantification Kit (Roche) was used to quantify the high-quality library and submit it for sequencing. Libraries for single-cell sequencing experiments were performed using the HiSeq4000. The raw sequencing data were checked and initially processed using Cell Ranger software (version 3.1.0).

### ScRNA-Seq Data Processing

Raw sequencing data were processed with the Cell Ranger pipeline (version 3.1.0) and mapped to the hg38 reference genome to generate matrices of gene counts by cell barcodes, as previously described. In brief, FASTQ files were aligned to the hg38 genome reference, and transcriptomes with Cell Ranger counts that used an aligner called STAR with default settings, and aligned reads were filtered for valid cell barcodes and UMI to generate filtered gene-barcode matrices. Then, unique molecular identifier (UMI) count matrices were imported into R (version 4.0.2) and processed with the R package Seurat (version 3.2.0) (11). We quantified the proportions of UMIs mapped to the mitochondrial genome, and the data were filtered to include genes detected in >5 cells, cells with 200–3000 detected genes, and UMI with 1000–15000 and <10% mitochondrial genes. Ultimately, we obtained 7,665 and 8,953 cells for further analysis.

For single-cell data analysis, ‘FindIntegrationAnchors’ and ‘IntegrateDatafunction’ were used to integrate two sample datasets and obtain the log-normalized expression value. Then, the ‘Scale Data’ function was used to scale the whole expression data. To identify the variable genes, the ‘FindVariableFeatures’ function was used. Principal component analysis (PCA) of the genes from these selected cells was conducted. The principal components (PCs) used to partition the cells were selected using JackStraw (1,000 replicates). The first 15 PCs were used to perform UMAP analysis using the ‘RunUMAP’ and ‘FindNeighbors’ functions. Finally, we used the function ‘FindClusters’ that implements the SNN (shared nearest neighbor) modularity optimization-based clustering algorithm on 15 PCA components with resolution 0.5 - 1.5, leading to 10-24 clusters, and a resolution of 1.2 was chosen for further analysis. The ‘FindAllMarkers’ function was used with the likelihood-ratio test for single-cell gene expression to identify marker genes. We performed DEG analysis by comparing each cluster between two groups of samples using the Wilcoxon rank-sum test, and genes with 1.2-fold changes and P values less than 0.05 were designated as significant signatures. We use ‘prop. test’ in R/4.0.2 when comparing cell type ratios. Bonferronis correction was used to correct the p-value calculated by prop.test to reduce false positives. Statistical significance was set at P <0.05. The basic metrics of scRNA-seq are listed in **Supplementary Table 1**, and information related to integrated quality control of scRNA-seq was shown in **Supplementary Figure 4**.

### ScATAC-Seq Data Processing

The original data of scATAC were obtained from previous experimental results of our research group (33). Sequencing data are available under the accession number GSE157595. We performed single-cell chromatin accessibility analysis integrated with scRNA-seq data using ArchR (51). The process was the same as that of scRNA-seq. In simple terms, after obtaining the original sequencing data, we used Cell Ranger ATAC (version 1.2.0) to compare with the Grch38 genome to generate sample-specific peak results. Furthermore, we used the ArchR software package in R software for further analysis and imported the filtered fragments. To obtain better data quality, we filtered the data using a minTSS of 4, minFrags of 1,000, and maxFrags of 100,000. In the end, we obtained 1,822 cells from NC-PBMCs and 4,977 cells from AS-PBMCs. The average TSS scores of the two samples were 12 and 15, respectively. For scATAC-seq data dimensionality reduction, we chose the default dimensionality reduction method ‘addIterativeLSI’ in the ArchR software package and finally counted 30 dim for dimensionality reduction. The software used Seurat’s method for clustering and finally determined that the clustering resolution was 1.5, and 11 cell clusters were obtained. To perform cell-type annotation more accurately, we used the ‘addGeneIntegrationMatrixfunction’ in the ArchR software package to assist annotation in predicting clustering of scATAC-seq data; then, we identified MonoDC cells based on atac data, which could not be identified when atac data were individually identified. Finally, we obtained 12 clusters from the ATAC data. For the difference analysis in the scATAC-seq data, we used the default method in the ArchR software, and used 1.2-Fold and p value <0.05. Two different peaks were selected for motif enrichment analysis. One is the difference peak between the AS and NC groups. The other is the cell-specific peak (marked peak) for motif enrichment analysis. ScATAC-seq data processing included genome alignment, peak analysis, clustering, and TF motif analysis. For more analysis results of ScATAC data, please refer to the ArchR official site (https://www.archrproject.com/bookdown/getting-started-with-archr.html).

### Locate Cell-Type-Specific Causal Genes for AS by scRNA-Seq

We used a meta-analysis to identify genes related to the pathogenesis of AS. This overlaps with the DEG obtained in this study to obtain cell-type-specific AS-causing genes. We entered search terms in the following databases PubMed, Cochrane Library, Embase, Web of Science, SpringerLink, CNKI, Embase, Cochrane and clinical trial [“ankylosing spondylitis” or “spondylitis, ankylosing”(MeSH) or “autoimmune”] and [“genes” or “Differentially expressed gene” (MeSH)] (the last search was updated on October 30, 2020) to identify relevant studies. The inclusion criteria were as follows: (a) case-control study; (b) study providing gene expression data; (c) study with ankylosing spondylitis patients diagnosed based on the modified New York criteria; and (d) further evidence that these DEGs are indeed involved in immune-related physiological and pathological processes. Two researchers evaluated the included study independently, and the third was assigned to evaluate the study with a large difference in scores. Quality assessment was performed using the Newcastle-Ottawa Scale (NOS). Three quality appraisal items were used in this meta-analysis, with scores ranging from 0 to 9. Scores–0-3, 3-6, and 6-9 were defined as low, moderate, and high quality, respectively. The final data collection was confirmed by a check between the two researchers. The odds ratios (ORs) and 95% confidence intervals (95% CIs) were calculated using Review Manager Version 5.1.6 (provided by the Cochrane Collaboration, available at: https://www.cochrane.org/) and STATA Version 12.0 (Stata Corp, College Station, TX, USA). Between-study variations and heterogeneities were estimated using the Cochran Q-statistic (52); P ≤ 0.05, was considered to represent statistically significant heterogeneity. Whenever a significant Q-test (P ≤ 0.05) indicated heterogeneity, a random-effects model was generated for meta-analysis. The CI was 95%. As a result, we conducted a meta-analysis of all 11 cohorts to assess the AS-related gene expression signatures. A total of 96 AS patients and 67 NCs were included in the sequencing data for differential gene expression meta-analyses. In this study, we used the INMEX (integrative meta-analysis of expression data) program (https://www.networkanalyst.ca/) (53) to carry out the meta-analysis. Meta-analysis showed that 107 DEGs in AS were statistically significant and involved in the pathophysiological process of AS. We overlapped these genes with the DEGs in our study. Associating AS-causing genes with specific cell types allows us to have a more three-dimensional and comprehensive understanding of how these genes participate in the occurrence and development of AS.

### Construction of the AS Casual Gene-Related Regulatory Network

Integration of scATAC-Seq and scRNA-Seq data used co-clustering-based unsupervised transfer learning. According to the related literature on AS pathogenic principles and the results of GO and KEGG analysis of the differentially expressed genes of each cluster, the disease-specific cell subgroups were selected, and the candidate target genes were selected from the differentially expressed pathogenic genes. Follow-up combined with the results of scATAC to further map out how the selected target genes participate in the pathogenesis of the AS regulatory network.

### Validation of key DEGs Using Immunomagnetic Bead Sorting and RT-qPCR

To further verify the DEGs in our study, we recruited three AS patients (male, mean age 24 ± 6 years, ASDAS-CRP>2.5) and three healthy volunteers matched by age and sex in the Department of Rheumatology, Shenzhen People’s Hospital, in June 2021. CD8+ T cells were positively selected by magnetic bead sorting with positive selection magnetic cell sorting (MACS) immunobeads (Miltenyi Biotec Inc., Bergisch Gladbach, Germany). RNA was extracted from CD8+ T cells using RNAiso Plus (TAKARA, 9109), chloroform, and isopropanol. Total RNA was reverse-transcribed into cDNA using PrimeScript RT Master Mix (Takara, RR036A). Key genes in our study, including *TNF*, *NFKB*, *FOS*, *JUN*, and *JUNB*, were chosen for validation of results by RT-qPCR.

The primer sequences for the genes were as follows: *NFKB* forward, TGCAGCAGACCAAGGAGATG, *NFKB* reverse, CCAGTCACACATCCAGCTGTC; *TNF* forward, GACTGGAGTTGGACGACGTTC, *TNF* reverse, GAAGAGGACCTGGGAGTAGATG; *JUN* forward, GAGAGCGGACCTTATGGCTAC, *JUN* reverse, GTGAGGAGGTCCGAGTTCTTG; *FOS* forward, GGAGGGAGCTGACTGATACAC, *FOS* reverse, AGCTGCCAGGATGACTCTAG; *JUNB* forward, CCCTGGACGATCTGCACAAG, *JUNB* reverse, GAGTAGCTGCTGAGGTTGGTG.

### Statistical Analysis

All statistical tests were performed using R/4.0.2. Data are presented as the mean ± SD of two independent experiments. Means were compared using Welch’s t-test or unpaired t-test. A P value < 0.05 was considered statistically significant.

## Data Availability Statement

ScATAC-seq data from patients and NC reported in this study have been deposited in NCBI Gene Expression Omnibus under the accession number GSE157595. Related website is: https://www.ncbi.nlm.nih.gov/geo/query/acc.cgi?acc=GSE157595; ScRNA-seq data have been deposited in Genome Sequence Archive for Human (https://ngdc.cncb.ac.cn/gsa-human/) under the accession number HRA001027.

## Ethics Statement

This study was approved by the Ethics Committee of Jinan University. The patients/participants provided their written informed consent to participate in this study.

## Author Contributions

XH and HY interpreted the data and wrote the main manuscript text. HW prepared all the figures. WC and CZ performed the experiments. XH contributed to the interpretation of data. LL and DL reviewed the manuscript. XH performed the data analysis. DT and YD supervised the experiments and contributed to the interpretation of data. All authors contributed to the article and approved the submitted version.

## Funding

This study was supported by grants from Shenzhen Key Medical Discipline Construction Fund(No.SZXK011), Guangxi Key Laboratory of Metabolic Diseases Research (20-065-76), the science and technology plan of Shenzhen(NO : JCYJ20180306140810282), Shenzhen Fund for Guangdong Provincial High-level Clinical Key Specialties (NO.SZGSP001) and Key Research and Development Program of Guangdong Province (Grant No. 2019B020229001).

## Conflict of Interest

The authors declare that the research was conducted in the absence of any commercial or financial relationships that could be construed as a potential conflict of interest.

## Publisher’s Note

All claims expressed in this article are solely those of the authors and do not necessarily represent those of their affiliated organizations, or those of the publisher, the editors and the reviewers. Any product that may be evaluated in this article, or claim that may be made by its manufacturer, is not guaranteed or endorsed by the publisher.
