# Supplementary material for: Integrative Single-Cell RNA-Seq and ATAC-Seq Analysis of Peripheral Mononuclear Cells in Patients With Ankylosing Spondylitis
Source: Front Immunol. 2021 Nov 22;12:760381. doi: 10.3389/fimmu.2021.760381 (PMC8647172; doi:10.3389/fimmu.2021.760381)
Supplement: Supplementary file 1 [file DataSheet_1.docx]

Supplementary Material

## Supplementary Figure


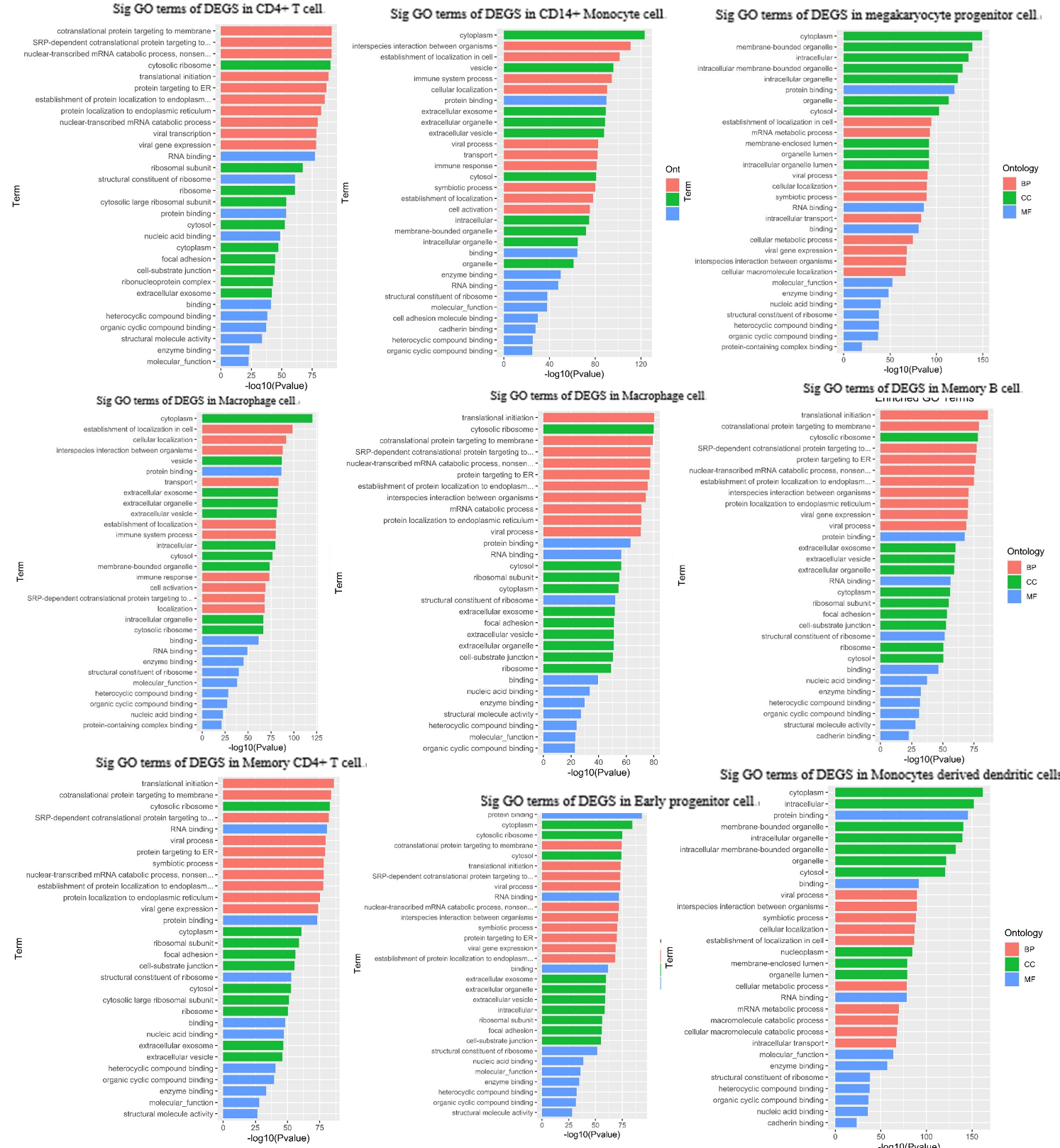


Supplementary figure 1. GO term analysis for biological processes of differentially differential genes between the AS-PBMC and NC-PBMC libraries in different cell type.


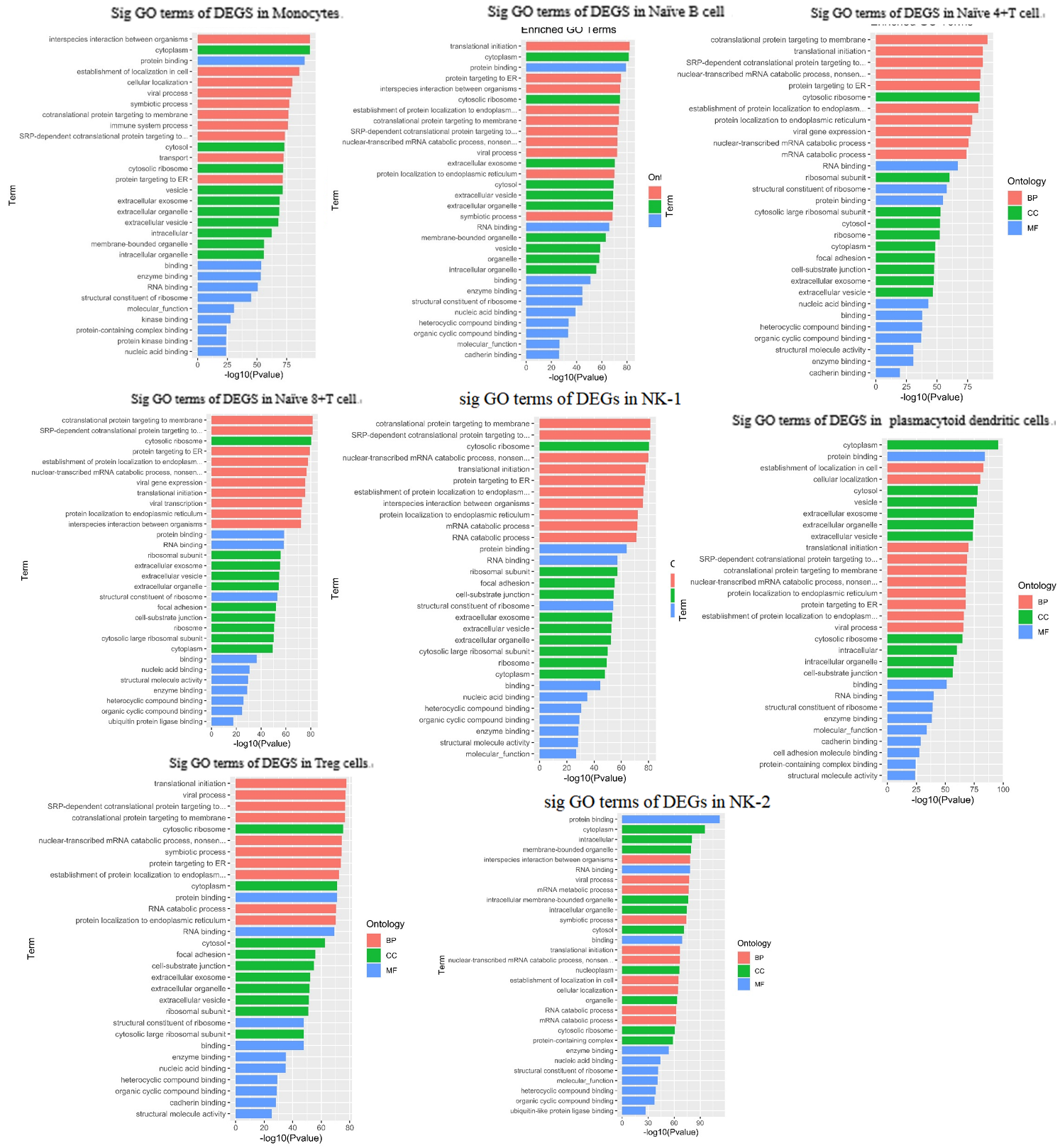


Supplementary figure 2. GO term analysis for biological processes of differentially differential genes between the AS-PBMC and NC-PBMC libraries in different cell type.


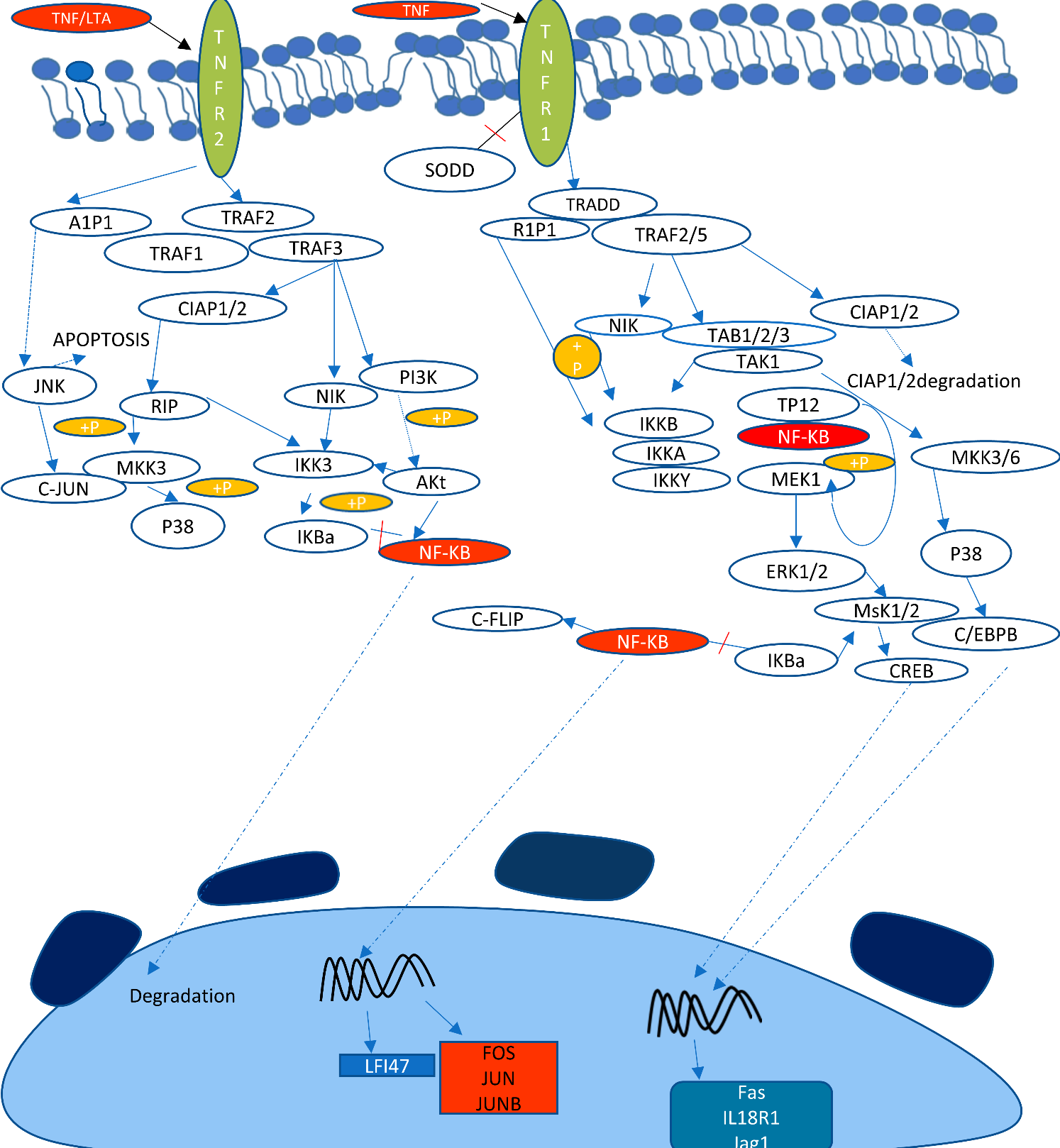


Supplementary figure 3.*TNF, NFKB, JUN, FOS* and *JUNB* involved in the TNF signaling pathways.


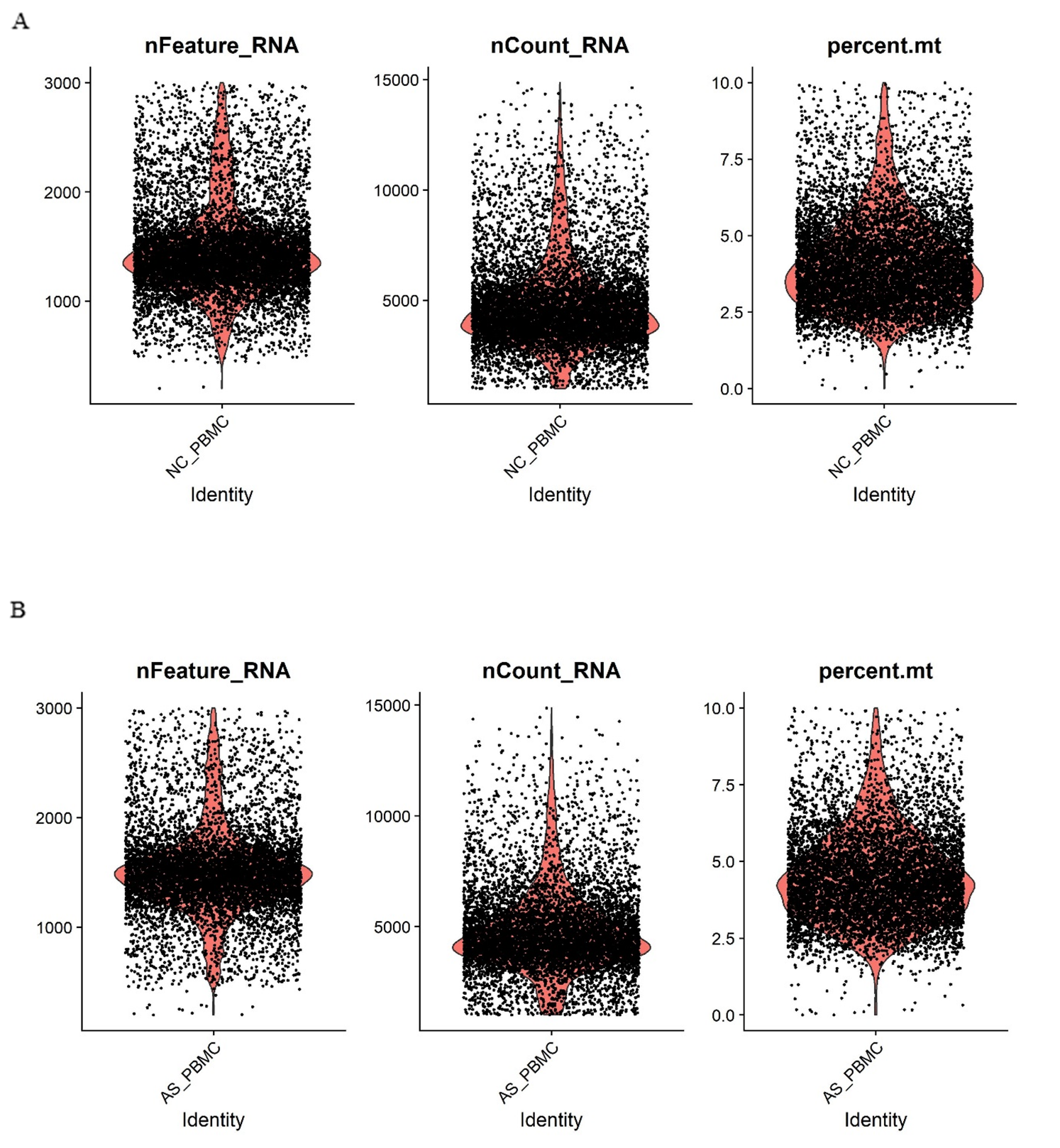


Supplementary figure 4. ScRNA-seq data after quality control and gene filtering.

1. Scatterplots illustrating the number of genes (left), unique molecular identifiers (UMIs) (middle) and the percentage of mitochondrial genes (right) in each cell of **NC** groups after quality control and gene filtering.
2. Scatterplots illustrating the number of genes (left), unique molecular identifiers (UMIs) (middle) and the percentage of mitochondrial genes (right) in each cell of **AS** groups after quality control and gene filtering.
3. **Supplementary Tables**

Supplementary table1 The basic metrics of scRNA-seq.

| Group | Number of cells  be obtained | Number of cells  be used | Mean reads  per cell | Median genes  per cell |
| --- | --- | --- | --- | --- |
| AS-PBMC | 8,821 | 7,665 | 45,830 | 1,446 |
| NC-PBMC | 10,372 | 8,953 | 36,221 | 1,354 |

Supplementary table 2 Summary of the cell type-specific markers be used in our study.

| Makers | Cell Type | Reference（ PMID or Web site address） |
| --- | --- | --- |
| CD4+  CD3D+  CD3D+  CD8+  CD8A+  CD8B+  CD8A+  CD8B+  CD4+  IL7R+  CD27+  S100A4+  CD4+  CD3D+  CCR7+  CD8B+  SELL+  GZMB+  GZMK+  CD3D+  CD8A+  TNFSF8+  CD8B+  CD4-  CD3D+  SLC4A10+  TRAV2+  FOXP3+  CD4+  CCR10+  IL7R+  CD52+  CMTM7+  NKG7+  TBX21+  GNLY+  CD247  CD14+  LYZ+  S100A12+  CD16+  S100A12+  CEBPB+  S100A8+  CD68+  CD14+  CD14+  CST3+  CD1C+  FCER1A+  NPP1+  IL10RA+  ITM2C+  GZMB+  CD1C+  CD80+  CD79A+  CD19+  CD24+  CD79A+  TCL1A+  CD19+  CD27+  CD14+  CD68+  MS4A7+  CSF1R+  PPBP+  PF4+  CD34+  ZNF683+  CD8A+  KCRB1+  NCAM1+ | CD4+T  CD4+T  CD8+T  CD8+T  CD8+T  CD8+T  memory CD4+T cell  memory CD4+T cell  memory CD4+T cell  memory CD4+T cell  memory CD4+T cell  memory CD4+T cell  naïve CD4+T cell  naïve CD4+T cell  naïve CD4+T cell  naïve CD4+T cell  naïve CD4+T cell  naïve CD4+T cell  naïve CD8+ T cell  naïve CD8+T cell  naïve CD8+T cell  naïve CD8+T cell  naïve CD8+T cell  naïve CD8+T cell  MAIT T  MAIT T  MAIT T  Treg  Treg  Treg  Treg  Treg  Treg  NK  NK  NK  NK  CD14 MONO  CD14 MONO  CD14 MONO  CD14 MONO  monocytes  monocytes  monocytes  monocytes  monocytes  MONO DC  MONO DC  MONO DC  MONO DC  PDC  PDC  PDC  PDC  PDC  PDC  naïve B  naïve B  naïve B  memory B  memory B  memory B  memory B  macrophage  macrophage  macrophage  macrophage  mk  mk  early pro  NK-2  NK-2  NK-2  NK-2 | 20868565；12000723  NA  28263960  28263960  29361178  29361178  NA  NA  29361178  29361178  29361178  NA  22697005  NA  28622514  22697005  22697005  NA  29361178  29230012  24119592  28622514  29361178  NA  NA  28622514  NA  28622514  29230012  29361178  28622514  29361178  29361178  28263960  <https://assets.thermofisher.com/TFS-Assets/LSG/brochures/immune-cell-guide.pdf>  29361178  30093597  29361178  29361178  29361178  29361178  30093597  30093597  30093597  25799053  25799053  29361178  29361178  29361178  29361178  NA  NA  12384430  29361178  <https://www.biolegend.com/cell_markers>  <https://www.biolegend.com/cell_markers>  23608739  23608739  NA  NA  NA  27780620  27780620  29096690  29230012  29262845  <https://www.biolegend.com/cell_markers>  29361178  29361178  25591800  29361178  <https://www.bio-rad-antibodies.com/human-immune-cell-markers-selection-tool.html#cell=nk-cells>  NA  29610856 |

“+”indicates higher expression, while “-” indicates lower expression; NA, not applicable.

# Supplementary table 3 General characteristics of AS group and healthy group.

| Characteristics | scRNA-seq scATAC-seq | | | | |
| --- | --- | --- | --- | --- | --- |
|  | AS | | NC | AS | NC |
| Number of cases  Mean Age, (years)*  Sex ratio (male: female)  Disease duration (years)*  HLA-B27 (+/-)  ASDAS-CRP* | | 6  27±3  6:0  7.04±2.66  6/0  1.21±0.42 | 6  26±3  6:0  NA  NA  NA | 9  37 ± 6  7:2  8.22 ±4.25  6/3  2.42±0.55 | 12  34 ± 9  6:6  NA  NA  NA |

ASDAS-CRP: CRP-based disease activity score; NA, not applicable. *Mean ± standard deviation.
